# Impedance cardiography as tool for continuous hemodynamic monitoring during cesarean section: randomized, prospective double blind study

**DOI:** 10.1186/s12871-018-0498-4

**Published:** 2018-03-27

**Authors:** Alessandro D’Ambrosio, Antonella Cotoia, Renata Beck, Potito Salatto, Lada Zibar, Gilda Cinnella

**Affiliations:** 1 Department of Anesthesia, Intensive Care and Pain Therapy, University of Foggia, University Hospital Foggia, Foggia, Italy; 2 Department of Pathophysiology, Faculty of Medicine, University of Osijek, Osijek, Croatia; 3 Institute for Nephrology, Osijek University Hospital, Osijek, Croatia

**Keywords:** Impedance Cardiography, Hemodynamics, Cesarean section, Levobupivacaine, Hypotension

## Abstract

**Background:**

Impedance Cardiography (ICG) is a non-invasive tool for continuous hemodynamic monitoring. Aims of our study were to assess the utility of ICG to evaluate the hemodynamic impact of 6 mg (GL6) vs 8 mg (GL8) levobupivacaine combined with fentanyl in healthy patients undergoing elective cesarean section; secondary, to compare the duration and quality of analgesia and anesthesia.

**Methods:**

Sixty-two women receiving combined spinal-epidural (CSE) for elective cesarean delivery were randomly allocated to GL6 or GL8 groups. Mean arterial pressure (MAP), cardiac index (CI), systemic vascular resistance index (SVRI), heart rate (HR), stroke volume index (SVI) were recorded from Tbaseline to 31 min after CSE by ICG. Sensory and motor blocks, patients and surgeons satisfaction, neonatal data were also recorded.

**Results:**

Fifteen of 32 patients in GL6 and 15 of 30 patients in GL8 experienced hypotension at T2 vs Tbaseline (*P* < .001) and SVRI reduction (*P* = .035 and P < .001 respectively). MAP, CI and SVRI were always slightly higher in GL6 vs GL8. HR and SVI remained stable until the end of surgery in all patients. Total ephedrine requirements was higher in GL8 (*P* = .010). The onset and offset time of sensory and motor block were similar in both groups, but the number of patients with motor block was lower in GL6 vs GL8 (*P* = .001). Patients and surgeon satisfaction scores, the number of patients needed systemic rescue doses, neonatal data were similar in both groups.

**Conclusions:**

ICG is a useful noninvasive tool to monitor continuously hemodynamics during cesarean section. The hemodynamic stability, the satisfying sensory block and rapid mobilization provided by low levobupivacaine dose may be particularly advantageous in obstetric patients.

**Trial registration:**

ClinicalTrials.gov: NCT03170427. Retrospectively Registered (Date of registration: May 2017).

## Background

Impedance Cardiography (ICG) has been proposed as a non-invasive method for hemodynamic monitoring of adult cardiopathic patients as well as for patients in the emergency department, wards and ambulatories [1]. ICG measures changes in resistance to electrical current proportional to blood flow from major vessels during each cardiac cycle, by four small sensors placed on the skin surface over the thorax. The ICG allows a reliable, simple and quick method to perform continuous monitoring of patient’s hemodynamic through calculation of a series of parameters, such as cardiac index (CI), stroke volume index (SVI), systemic vascular resistance index (SVRI), in addition to non-invasive blood pressure and heart rate (HR) [2, 3].

Maternal hemodynamic changes during spinal anesthesia for cesarean section are traditionally evaluated by HR and non-invasive blood pressure but recent studies addressed the importance of cardiac output (CO) monitoring in the assessment of the maternal hemodynamic stability [2, 4, 5]. Some studies reported the validity of ICG to monitor the effect of pharmacological therapy in pregnancies at risk for hypertensive complications [6]. Recently Morris et al. studied the impedance cardiography values either during normal pregnancy and postpartum after vaginal or cesarean delivery [3], while Tihtonen used ICG during the course of caesarian section in pre-eclamptic parturients [2]. However, to our knowledge, no data are available on impedance measurement during spinal anesthesia with different local anesthetic doses whereas several studies were focused on the optimal dose of local anesthetics to obtain a good anesthesia and reduce the motor block [7, 8].

The purpose of this prospective randomized double-blind study was thus to assess the utility of ICG to evaluate the hemodynamic impact of 6 mg vs 8 mg levobupivacaine combined with fentanyl in healthy patients undergoing elective cesarean section; a secondary aim was to compare the duration and quality of analgesia and anesthesia.

## Methods

After ethical approval, this prospective randomized, double blinded study was performed in the Obstetrics and Gynecology Department of University Hospital of Foggia, Italy from 1 January 2013 to 31 May 2014. Written informed consent were obtained during the preoperative visit performed by the investigators (RB, PS). All consecutive healthy women, between 18 and 45 years, undergoing elective Caesarean delivery at term of singleton pregnancy, with American Society of Anesthesiologists physical status of class I or II, without preeclampsia and diabetes, without a history of abdominal surgery, 155–180 cm in height, were considered for enrollment. Patient with a known allergy to amide local anesthetics and other drugs, with body mass index (BMI) ≥40 kg/m^2^, cardiologic or systemic disease, in treatment with antihypertensive or anticoagulant were excluded. On arrival in the recovery room the ICG non-invasive blood pressure cuff was placed on the left arm, two sensors were placed above the clavicle on each side of the neck, and two sensors were placed on either side of the thorax at midaxillary line corresponding to the level of the xiphoid process. The ICG algorithm uses thorax impedance change to calculate CI, SVI, SVRI, HR while mean arterial pressure (MAP) is measured by oscillometric method (Philips Medical Systems 3000 Minuteman Road Andover, MA, Nederland). These parameters were recorded at baseline, defined as the average of the first 5 min before neuraxial anesthesia (T0), every minute after neuraxial block for 10 min (T1-T10) and then every 3 min for 20 min (T10-T31). Arterial pulse oxygen saturation (SpO2) and five-lead ECG were applied as perioperative routine monitoring (Philips IntelliVue™ Monitoring; Philips Medical Systems, Andover, MA, USA). Intravenous crystalloid pre-loading (lactated Ringer’s solution, 10 ml/kg) was infused over 10 min prior to lumbar puncture. Thereafter, co-loading of 10 ml/kg/h lactated Ringer’s solution was administered [9]. Using a computer- generated sequence of numbers, patients were randomly allocated in one of the two groups: 6 mg (1.6 mL) levobupivacaine + 20 μg fentanyl (GL6 group) or 8 mg (2 mL) levobupivacaine + 20 μg fentanyl (GL8 group). Continuous spinal epidural anesthesia (CSE) was performed with patient in sitting position: a 18-gauge Tuohy needle was inserted into the L2-L3 interspace using the loss of resistance of saline technique to identify the epidural space; a 27-gauge Withacre spinal needle was then placed through the Tuohy needle until the dura mater was punctured and isobaric undiluted levobupivacaine plus 20 μg fentanyl was administered. Afterwards, an epidural catheter (Espocan, B.Braun, Melsungen, Germany) was inserted 4 cm into the epidural space. No test dose with lidocaine was given. Patients were immediately positioned supine and uterus was manually displaced to the left. To facilitate blinding, central blocks execution, syringes preparation and data recording were performed by three different anesthesiologists (AD, PS, RB respectively).

Sensory levels were checked using ice test cold and touch with alcohol puffs, and motor block was measured by modified Bromage scale (0 = no paralysis and able to flex hips/knees/ankles; 1 = able to move knees, unable to raise/extend legs; 2 = able to move feet only; 3 = unable to move any part of the lower limb) [10–12]. Both tests were performed every 5 min from T1 until delivery, then at 15 min intervals until the sensory variables receded to dermatome T12 and motor variables were back to normal. The onset time of sensory block was considered as the time interval between administration of local anesthetic and loss of touch and cold above T5. If the sensory block did not reach the T5 within 15 min the epidural top-up with mepivacaine 2% (Carbosen, Galenica Senese S.r.l.,Via Cassia Nord, 351, Monteroni D’Arbia, Italy) was given and the patient was dropped out from the study. The onset time of motor block was defined as the interval between intrathecal administration and a Bromage score of 1. The offset time of sensory or motor block was defined as the interval from intrathecal administration to the T12 dermatome regression time or to the point at which the Bromage score returned to zero. Patients were asked to report any intraoperative pain or discomfort using Numeric Rating scale (NRS) of 0–10. If the patient reported discomfort or NRS ≥3 when parietal peritoneum or the muscles fascia or the skin were closed, fentanyl 50 μg and midazolam 1 mg were administered intravenously as a rescue dose.

Hypotension, defined as a threshold of MAP≤65 mmHg, was treated immediately with ephedrine 3 mg IV. Bradycardia, defined as reduction in HR > 20%, was treated by atropine 0.5 mg IV.

Nausea and vomiting were treated with ondansetron 4 mg IV. Thirty seconds after delivery, 5 U oxytocin in 10 ml water was administered over a period of 60 s. Fetal presentation, birth weight, time to birth, the duration of surgery, the postoperative NRS measured 15 min after surgery, were also recorded. Newborns were evaluated with Apgar scores at 1 min and 5 min and umbilical cord blood gas values. At the end of the surgery, patients and surgeons satisfaction was assessed using a descriptive 3-point verbal rating scale (3 extremely satisfied; 2 satisfied; 1 not satisfied) [4]. We used epidural catheter for postoperative analgesia with elastomeric pump (200 mg levobupivacaine and 100 μg fentanyl diluted with saline solution at 5 mL/h in 48 h).

### Statistical analysis

The power analysis suggested that a sample size of 21 parturients/group was required to detect a decrease of blood pressure > 25% from the baseline(assuming α = .01 and power = .95) [13] and that a sample size of 28 patients/group was required to detect a 30 min difference in motor block duration (assuming α = .01 and power = .95) [4]. The number was then increased to 33 per group to allow for a 20% patients drop-out rate.

The normality of distribution was assessed by Shapiro-Wilkinson test. Since we found almost all of the data normally distributed, the data were expressed as mean ± SD values or number. Data were analyzed using repeated measurements analysis of variance (RANOVA). Differences between the groups at each time point were examined post hoc using independent sample t-test. A paired sample t-test was used to detect changes within the groups. Level of statistical significance was chosen to be at *P* < .05. Statistical analysis was performed by Statistical Package for the Social Sciences (SPSS Inc., Chicago, IL) version 15.0 for Windows.

## Results

**Fig. 1:**
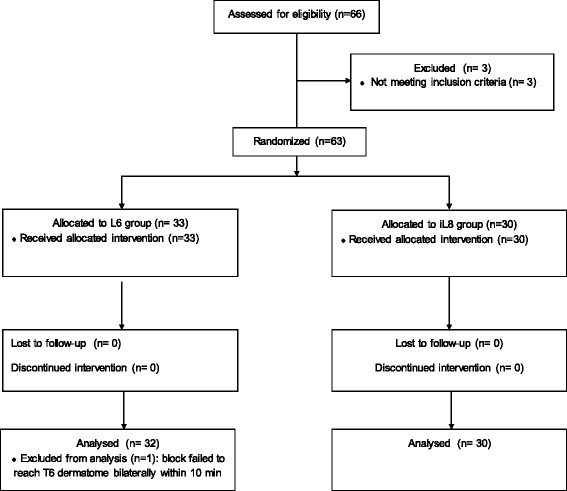
Flow chart of patients’ enrolment

The enrolment flow diagram is reported in Fig. 1. Sixty-two out of 66 patients candidate for enrolment were included in the study. There were no differences between the two groups as regards age, height, weight, BMI, gestational age, fetal presentation, baseline hemodynamic data and surgery duration (from surgical incision to skin closure) (Table 1).

**Table 1:** Demographic and baseline hemodynamic data

	6 mg Levobupivacaine (*n* = 32)	8 mg Levobupivacaine (*n* = 30)	*P* value
Age (years)	34 ± 5	32 ± 5	.235
Height (cm)	163 ± 6	164 ± 5	.985
Weight (kg)	78 ± 12	83 ± 14	.978
BMI (kg/m^2^)	29.5 ± 3	30 ± 4	.696
Gestational age (weeks)	38 ± 1	38 ± 1	.993
Cephalic/ breech presentation (n)	28/4	27/3	.756
MAP (mmHg)	93 ± 8	96 ± 13	.248
HR (bmp)	96 ± 15	100 ± 13	.129
CI (L/min/m^2^)	3.5 ± .6	3.7 ± .6	.310
SVRI (dyn-sec/cm^5^/m^2^)	1812 ± 336	1789 ± 363	.704
SVI (mL/beat/m^2^)	41.2 ± 5	41 ± 6	.715
Duration of surgery (min)	55 ± 15	55 ± 11	.973

BMI = Body mass index, MAP = mean arterial pressure, HR = heart rate, bmp = beats per minute, CI = cardiac index, SVRI = systemic vascular resistance index, SVI = stroke volume index

Data are expressed as mean ± standard deviation or number

Baseline values are defined as average of the first 5 min of measurements before neuraxial anesthesia

### Effects of local anesthetic on hemodynamic

**Fig. 2:**
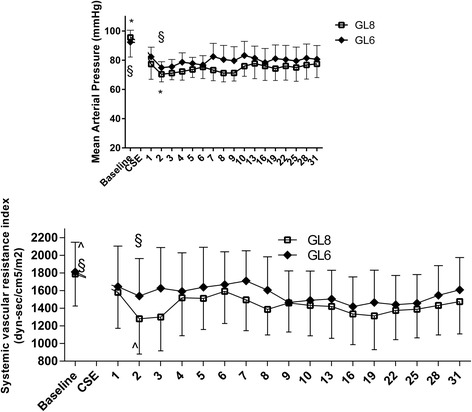
Mean arterial pressure (mmHg) and systemic vascular resistance index (dyn-sec/cm^5^/m^2^) trend. Data are presented as mean in fifteen patients in GL6 group and fifteen patients in GL8 group. * § *P* < .001; ^ *P* = .035

**Fig. 3:**
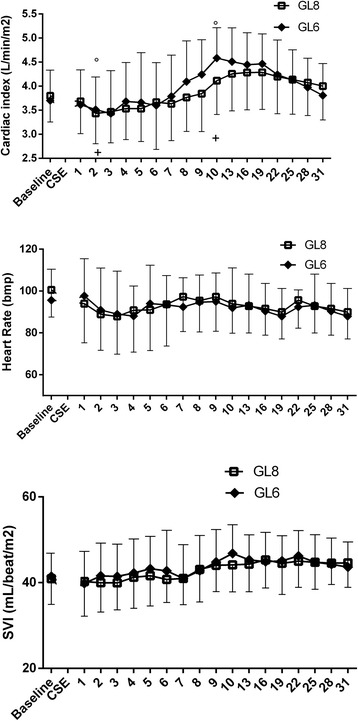
Cardiac index (L/min/m^2^), heart rate (bpm) and SVI (mL/beat/m2) trends. Data are presented as mean in fifteen patients in L6 group and fifteen patients in L8 group. ° *P* = .003; + *P* = .052

In 32 patients (17 from GL6 and 15 from GL8), there were no statistically significant hemodynamic changes after spinal anesthesia and throughout the study period. Therefore we present the analysis of 30 patients with post spinal hypotension (MAP ≤ 65 mmHg). On T2 fifteen patients (47%) in GL6 and fifteen patients (50%) in GL8 experienced hypotension (*P* < .001 vs Tbaseline) and SVRI reduction (*P* = .035 and P < .001vs Tbaseline respectively). After the ephedrine administration, MAP and SVRI returned in the normal range in every patient and remained stable (Fig. 2). CI slightly decreased on T2 by 8% in GL6 and by 17% in GL8 (*P* = .721 and *P* = .125 vs Tbaseline respectively). CI was 28% higher on T10 vs T2 in GL6 (*P* = .003) and 24% higher in GL8 (*P* = .052). Thereafter, CI progressively returned to the baseline value. HR and SVI remained stable until the end of surgery in all patients (Fig. 3).

Intergroup comparison showed that MAP, CI and SVRI were higher, though not significantly, in GL6 vs GL8 during the whole study period. Total ephedrine requirements was 3.4 ± 3.3 mg in GL6 vs 5.7 ± 3.5 mg in GL8 (*P* = .010). Time to birth, total amount of liquids, percentage of nausea/vomiting, shivering and pruritus and postoperative NRS were similar in both groups (Table 2). Patients and surgeons reported high satisfaction in both groups (Table 2).

**Table 2:** Intraoperative data

	6 mg Levobupivacaine (*n* = 32)	8 mg Levobupivacaine (n = 30)	*P* value
Patients with hypotension (n,%)	15 (47%)	15 (50%)	.012
Ephedrine (mg)	3.4 ± 3.3	5.7 ± 3.5	.010
Fluids (ml/kg)	19 ± 6	17 ± 5	.075
Shivering n (%)	5 (16%)	3 (10%)	.948
Pruritus n (%)	2 (6%)	1 (3%)	.951
Nausea/vomiting n (%)	7 (22%)	9 (30%)	.917
Time to birth (min)	8.8 ± 2	9.1 ± 2	.429
Intravenous rescue dose (n, %)	5 (15.6%)	3 (10%)	.357
Patient satisfaction score	2.5 ± .6	2.6 ± .6	.618
Surgeon satisfaction score	2.8 ± .4	2.9 ± .3	.628

Data are expressed as mean ± standard deviation or number of patients and percentage as appropriate

Patient and surgeon satisfaction was assessed using a descriptive 3-point verbal rating score (3 = extremely satisfied; 2 = satisfied; 1 = not satisfied)

Intravenous rescue dose = 50 μg fentanest + 1 mg midazolam

### Effect of local anesthetic on sensory and motor block

Sensory block was reached in 3.2 ± 1.4 and 2.8 ± .9 min respectively in GL6 and GL8 (*P* = .841). Maximal sensory block height (*P* = .999), offset time of sensory block (*P* = .735), regression time of 2 dermatomes (*P* = .547) and the number of patients needed systemic rescue dose were similar in both groups (*P* = .357) (Table 2-3). Nine patients in GL6 and 24 patients in GL8 experienced motor block (*P* = .001) with no differences in onset and offset times (*P* = .228 and *P* = .556 respectively) (Table 3).

**Table 3:** Sensory and motor block characteristics

	6 mg Levobupivacaine (n = 32)	8 mg Levobupivacaine (n = 30)	P value
Maximal sensory block height (n)			
T1 dermatome	27	23	.999
T2 dermatome	2	0	.999
C8 dermatome	3	7	.999
Onset time of sensory block (min)	3.2 ± 1.4	2.8 ± .9	.841
Offset time of sensory block (min)	157.2 ± 42	163.6 ± 33	.735
Regression time of 2 dermatomes (min)	62.4 ± 20	58 ± 14	.547
Motor block			
Bromage score 0 (n,%)	23 (71.9%)	6 (18.7%)	.001
Bromage score 1 (n, %)	4 (12.5%)	7 (23.3%)	
Bromage score 2 (n, %)	5 (15.6%)	14 (46.7%)	
Bromage score 3 (n, %)	0 (0%)	3 (10%)	
Onset time of motor block (min)	1.2 ± .4	1.9 ± 1.6	.228
Offset time of motor block (min)	60 ± 18	57 ± 7	.556

Data are presented as mean ± standard deviation or number and percentage of patients

### Neonatal data

There were no significant differences between the two groups as regards of birth weight, Apgar scores and umbilical cord acid-base measurements (Table 4).

**Table 4:** Neonatal demographics, Apgar scores and umbilical cord acid-base measurements

	6 mg Levobupivacaine (n = 32)	8 mg Levobupivacaine (n = 32)	P value
Apgar scores			
1 min	8 ± .9	8 ± 1	.656
5 min	9 ± .5	9 ± .8	.849
Umbilical vein			
pH	7.4 ± .1	7.4 ± 0	.965
PvO_2_ (mmHg)	25 ± 9	27 ± 5	.736
PvCO_2_ (mmHg)	42 ± 4	40 ± 5	.446
Base excess (mmol/L)	−1.8 ± 1	−1.9 ± .6	.941
Umbilical artery			
pH	7.3 ± 0	7.3 ± 0	.948
PvO_2_ (mmHg)	14 ± 5	11 ± 5	.724
PvCO_2_ (mmHg)	54 ± 5	54 ± 6	.952
Base excess (mmol/L)	−2 ± .8	−2.1 ± .8	.955
Birth weight (g)	3336 ± 381	3306 ± 365	.751

Data are presented as mean ± standard deviation

## Discussion

The main results of our study are: a) ICG was useful to perform a continuous hemodynamic monitoring and to detect hypotensive events in healthy patients undergoing cesarean section; b) 6 mg of intrathecal levobupivacaine produced a good sensory block with low incidence of motor block during the whole procedure, with a trend of hemodynamic values slightly higher as compared to 8 mg of levobupivacaine.

Pregnancy is a dynamic process associated with increased fetal and maternal metabolic demand and significant physiological changes in the cardiovascular system aimed at ensuring adequate uteroplacental circulation for fetal growth [14]. Therefore, maternal cardiac function monitoring, which more directly reflects uteroplacental perfusion changes, would be crucial in the hemodynamic management of parturients undergoing cesarean section to evaluate placental perfusion [15]. However, the ideal hemodynamic monitoring in healthy parturient should be non-invasive, with low or no potential side-effects and an high patients’ tolerance, since in this specific context theoretical considerations on devices safety and user-friendliness together with patients-bound factors, such as the need for further vascular accesses, can be barriers to a routine implementation of hemodynamic monitoring because the less sicker is the patients, the greater likelihood that benefit/risk ratio is perceived as unbalanced towards risks [16].

The novelty of the present study is the continuous real-time multiparametric hemodynamic monitoring during surgery obtained by ICG. ICG is a safe, simple to use, cost-effective and reproducible method that has been recently proposed as a valid alternative to the gold standard echocardiography to evaluate hemodynamics especially when the patient is the reference for himself [1, 17]. However, to our knowledge, ICG has not been extensively used in the obstetric field, although its validity to monitor cardiac function during pregnancy and in different maternal positions has been reported [17]. Recently Morris et colleagues confirmed that ICG, recorded in healthy pregnant women at specific times during gestation and postpartum, can be used to establish normative values useful to assess cardiopathic pregnant patients [3], while only one study, to our knowledge, used ICG to monitor hemodynamics *during* Cesarean section performed under spinal anesthesia with 0.5% hyperbaric bupivacaine [2]. Even if cesarean section is a matter of minutes in experienced hands, maternal hemodynamic instability may occur, especially in pre-eclamptic women: actually, on one side regional anesthesia may induce hypotension due to marked peripheral vasodilatation and reduced venous return only partially compensated by CI [18], on the other side after newborn’s delivery blood squeezed into systemic circulation by uterine involution and blood pooled from lower extremities do cause a preload increase and therefore a raise in CI [2]. Our data show that hypotension due to the sympathetic block-related reduction in SVRI occurred in about 50% of parturient; hypotension was only partially compensated by HR and therefore CI remained stable after the block. An increase in CI was observed only after newborn delivery, due to the above-mentioned preload mechanisms. This is confirmed by the finding that in non-hypotensive women CI remained stable after the block and increased only after delivery. In literature the reported incidence of hypotension after neuraxial block varies between 7.4% and 74.1% [18]. This wide range is due to differences in clinical setting studied but also to differences in the definition itself of hypotension. A clear-cut and widely accepted definition of hypotension after spinal anesthesia is currently lacking: however, even minor differences in definitions may cause major differences in the reported incidence, in the statistical power and last but not least in the evaluation of measures to prevent or treat hypotension itself. In the present study the physiological rationale for the strict cut-off value of MAP ≥65 mmHg allowed to identify the patients with a severe hemodynamic impairment near the threshold to maintain adequate tissue perfusion, below which any further disturbance may lead to serious complications [19].

The ICG devices studied in literature, measure blood pressure either by oscillometric method or by calculated method with the application of ICG cuffless technology [3, 20]. Herein, we used an ICG device measuring the MAP by the standard oscillometric technique.

Traditionally, three methods have been used for the prevention and management of spinal anesthesia-induced hypotension: fluid therapy, vasopressors and local anesthetic dosage [21]. Large volume preloading has been widely used as a strategy to prevent hypotension [22]. Actually, since preloading may results in extravascular fluid redistribution that dumps the hemodynamic effect of volume challenge [23], fluid co-load just following spinal anesthesia was proposed to reduce the incidence of hypotension with contrasting findings [24]. Recent evidence suggests to administrate half of the solutions’ amount as a pre-loading followed by co-loading, to manage spinal-induced hypotension and to reduce perioperative fluids volume [9]. Herein, we infused a pre-load of 10 ml/kg followed by a co-load of 10 ml/kg/h, thus avoiding aggressive intravascular volume challenge. Moreover, our data showed a lower incidence of intraoperative nausea and vomiting as compared to the associated maternal hypotension incidence, probably due to the strict blood pressure control by ICG that allowed the attending physician to promptly administer ephedrine boluses as needed.

In literature, there is widespread variation in the choice of administration of vasopressors in obstetric anesthesia, with ephedrine and phenylephrine acknowledged by the National Institute for Health and Care Excellence to be equally efficacious to counteract hypotension in healthy parturients. Although phenylephrine is claimed to be more preferable because of improved fetal acid-base status [25], there is lack of evidence showing differences with ephedrine when comparing the Apgar scores [26]. In our study, ephedrine boluses administered under strict hemodynamic control were efficacious in treating maternal hypotension, while no effects were observed in newborns, whose Apgar score 1 min after birth was always ≥8.

During spinal anesthesia blood pressure is inversely related to local anesthetics dose [27, 28] that can thus play a key role in the above mentioned hemodynamic effects. Therefore, in literature local anesthetics dose has been widely studied in order to find out the right matching between blood pressure reduction and perioperative analgesia quality [29–31].

As regards of the maximum level and the duration of sensory block, our results indicate that there was no difference between intrathecal administration of 6 mg vs 8 mg levopubivacaine 0,5% in combination with 20 μg of fentanyl. This may be because spinal anesthesia was performed at the L2-L3 interspace which has been shown to provide an adequate sensory block as compared to neuraxial anesthesia administered in lower interspaces [32]. With respect to motor block, its incidence was higher in GL8 than GL6, although the onset and offset time were similar. Providing no motor blockade offers the significant advantage of faster mobilization and more patient satisfaction [4].

Finally, the number of patients needing intravenous rescue doses during the closure of the parietal peritoneum or the muscles fascia or the skin was similar in both groups without affecting the maternal and surgeon satisfaction scores, which were high in both GL6 and GL8.

In literature, it was recently suggested to use a CSE technique when the dose of levobupivacaine is < ED_95_, in order to perform a prompt rescue in case of insufficient anesthesia [8]. However, our patients never need a supplemental analgesia through the epidural catheter which was placed under CSE technique.

There are several limitations of this study. First, patients were placed in supine position immediately after spinal block, whereas leg elevation significantly influenced the hemodynamic variables compared to the control group [33]. However we combined the supine position with left uterine displacement that decreases the incidence of hypotension [34, 35].

Another limitation of the study is that we recorded only indexed parameters. Whereas several studies measured the CI [2, 36], more recent results indicated a low correlation of CO with the body surface area in the obstetric patients [37].

## Conclusions

Our results suggest that ICG is a useful noninvasive tool to perform continuous hemodynamic control and to manage decisions in healthy parturients undergoing spinal anesthesia for cesarean section. Levobupivacaine 6 mg can be used safely for intrathecal anesthesia in obstetric patients providing a good hemodynamic stability, satisfactory intraoperative analgesia and rapid mobilization. Future objectives are the application of ICG in pregnant with cardiac disease undergoing urgent cesarean delivery.

## Abbreviations

CI: Cardiac index
CSE: Rate combined spinal-epidural
HR: Heart
ICG: Impedance Cardiography
MAP: Mean arterial pressure
NRS: Numeric Rating scale
SVI: Stroke volume index
SVRI: Systemic vascular resistance index
